# Latest trends in ADHD drug prescribing patterns in children in the UK: prevalence, incidence and persistence

**DOI:** 10.1136/bmjopen-2015-010508

**Published:** 2016-05-20

**Authors:** Raphaelle Beau-Lejdstrom, Ian Douglas, Stephen J W Evans, Liam Smeeth

**Affiliations:** 1 Institute of Global Health, University of Geneva, Geneva, Switzerland; 2 London School of Hygiene and Tropical Medicine, London, UK

**Keywords:** Medication use, Medication trends, attention deficit and hyperactivity disorder, paediatric mental health, attention deficit and hyperactivity disorder drugs

## Abstract

**Objectives:**

To investigate attention deficit and hyperactivity disorder (ADHD) drug prescribing in children under 16 years old in the UK between 1992 and 2013.

**Methods:**

All patients under 16 registered in the Clinical Practice Research Datalink (CPRD) with a minimum of 1 year of observation time and who received at least one prescription of any ADHD drug between 1 January 1992 and 31 December 2013.Trends in prevalence and incidence of use of ADHD drugs in children were calculated between 1995 and 2013 and persistence in new users was estimated.

**Results:**

The prevalence of ADHD drug use in children under 16 increased 34-fold overall, rising from 1.5 95% CI (1.1 to 2.0) per 10 000 children in 1995 to 50.7 95% CI (49.2 to 52.1) per 10 000 children in 2008 then stabilising to 51.1 95% CI (49.7 to 52.6) per 10 000 children in 2013. The rate of new users increased eightfold reaching 10.2 95% CI (9.5 to 10.9) per 10 000 children in 2007 then decreasing to 9.1 95% CI (8.5 to 9.7) per 10 000 children in 2013. Although prevalence and incidence increased rather steeply after 1995, this trend seems to halt from 2008 onwards. We identified that 77%, 95% CI (76% to 78%) of children were still under treatment after 1 year and 60% 95% CI (59% to 61%) after 2 years.

**Conclusions:**

There was a marked increase in ADHD drug use among children in the UK from 1992 until around 2008, with stable levels of use since then. UK children show relatively long persistence of treatment with ADHD medications compared to other countries.

**Strengths and limitations of this study**

- This study is providing an accurate and recent estimate of the patterns of use of attention deficit and hyperactivity disorder drugs in children during the past decades in the UK.
- The Clinical Practice Research Datalink (CPRD) has major strengths in the study of the use of medication in children. It allows for a recent, large, representative longitudinal cohort of children observing current UK practice.
- In this study, prescribing issued from general practitioners is described and not actual drug dispensation or consumption by the patient. However, a repeat of the prescription suggests the use of medication in the UK healthcare system.

## Introduction

Attention deficit and hyperactivity disorder (ADHD) is an early onset neurodevelopmental disorder combining overactivity and impulsivity with the inability to concentrate (The International Classification of Diseases (ICD) 10th edition criteria).^1^ Its worldwide prevalence was recently estimated at 5.29% in children.^2^ The consequences of ADHD can seriously impact quality of life affecting social behaviour and health of the child but also affect family relationships.^3^ Common therapeutic options include parental training, behavioural therapies and medication. Used since the 1960s, medications for ADHD are currently part of the “WHO list of essential medicines for common psychiatric disorders” which sets standards for medicines worldwide, including use in children. Methylphenidate and dexamphetamine were the first stimulant medications with proven efficacy in the treatment of core ADHD symptoms.^4^ Increase in the use of ADHD drugs has been observed in many studies worldwide.^5^ ^6^ During the past decade, new guidelines were issued, new pharmacological options were released and suspicions of serious cardiac long-term effects were raised.^7^ This study aims to give an overview of the current use of ADHD medications in children under 16 and its recent evolution in the UK. Our work describes the prevalence and incidence of ADHD drug use in children in the UK and estimates their persistence on treatment.

## Methods

### Data source: The Clinical Practice Research Datalink (CPRD)

The Clinical Practice Research Datalink (CPRD, formerly GPRD) is one of the world's largest databases of anonymised longitudinal medical records from primary care since 1987. The database includes data from UK GP practices that provide high quality data on more than five million active patients.^8^ The data collection is performed online and its completeness is assessed on a regular basis with routine checks to ensure data integrity and quality.^9^ Practices can only contribute data if they pass checks on quality and then are classified as ‘Up-to-Standard’ (UTS) for research purposes. The data cover more than 8% of the population and have been shown to be broadly representative of the UK population in terms of demographic characteristics when compared with the census in 2001.^10^ The validity of the CPRD has been investigated in many studies demonstrating the quality of the data available.^11–13^

### Selection of the patients

All patients registered in the CPRD for at least 1 year in a practice with UTS data and receiving at least one prescription of an ADHD drug (see list below) before the age of 16 between 1 January 1992 and 31 December 2013 were included. For reasons of anonymity, the CPRD database does not give exact date of birth. Only the year (or sometimes the month) of birth is available for all so age estimation is imprecise. Drugs of interest are those included in the British National Formulary chapter 4.4 “CNS stimulants and drugs used for attention deficit disorder”: methylphenidate (methylphenidate ‘immediate release’ and methylphenidate ‘extended release’), atomoxetine, dexamphetamine and modafinil.

### Statistical methods

Yearly prevalence was calculated dividing the number of children under 16 receiving at least one prescription of an ADHD drug during a year by the mid-year counts of children under 16 registered in the database during that year. Children were considered to receive a first prescription if they had a minimum of 12 months without any ADHD drug prescription after registration in the database or were aged below one. Yearly incidence of ADHD drug initiation used the number of children under 16 receiving a first prescription divided by the mid-year counts of children under 16 registered in the database during that year. A Wilcoxon type non-parametric test for trend was computed to investigate prescribing patterns over time.^14^

We also estimated persistence, defined as “the duration of time from initiation to discontinuation of therapy” according to Cramer *et al*.^15^ We therefore measured persistence considering first prescription to first discontinuation for each patient. First discontinuation of therapy was defined as the first gap of at least 90 days with continuous registration in the CPRD between the end of a prescription supply (approximated by the date of last prescription+30 days) and the next prescription. All participants were censored at the time of their last record in the CPRD. This analysis was also performed differentiating three groups (by age at first prescription): under 6 years old, 6–10 and 11–15. These were chosen to distinguish early use (often off-label use under 6 years for atomoxetine and methylphenidate and under 3 years for dexamphetamine), child use (6–10 years) and early or preadolescent use (11–15 years). The Kaplan-Meier estimator was used for persistence rates. After checking for proportional hazards, Cox regression was used to compare retention in each age group to the baseline (6–10 years). Statistical analysis used Stata Statistical Software: Release 13.( College Station, Texas: StataCorp LP, 2013.)

## Results

In our study, 14 748 children under 16 (85% boys) received at least one prescription of an ADHD drug between 1 January 1992 and 31 December 2013. Methylphenidate is by far the most used drug in children with ADHD in the UK accounting for 94% of ADHD prescriptions followed by atomoxetine, dexamfetamine and modafinil (table 1).

**Table 1:** Ranking of ADHD drug use in children under 16 years old, N=14 748: children could receive more than one type of ADHD drug

Drug substance name	Number of children (%)
Methylphenidate	13 908 (94.3)
Atomoxetine	2392 (16.2)
Dexamphetamine	581 (3.9)
Modafinil	27 (0.2)

ADHD, attention deficit and hyperactivity disorder.

### Trends in prevalence and incidence of ADHD drug prescription in children under 16 in the UK

**Figure 1:**
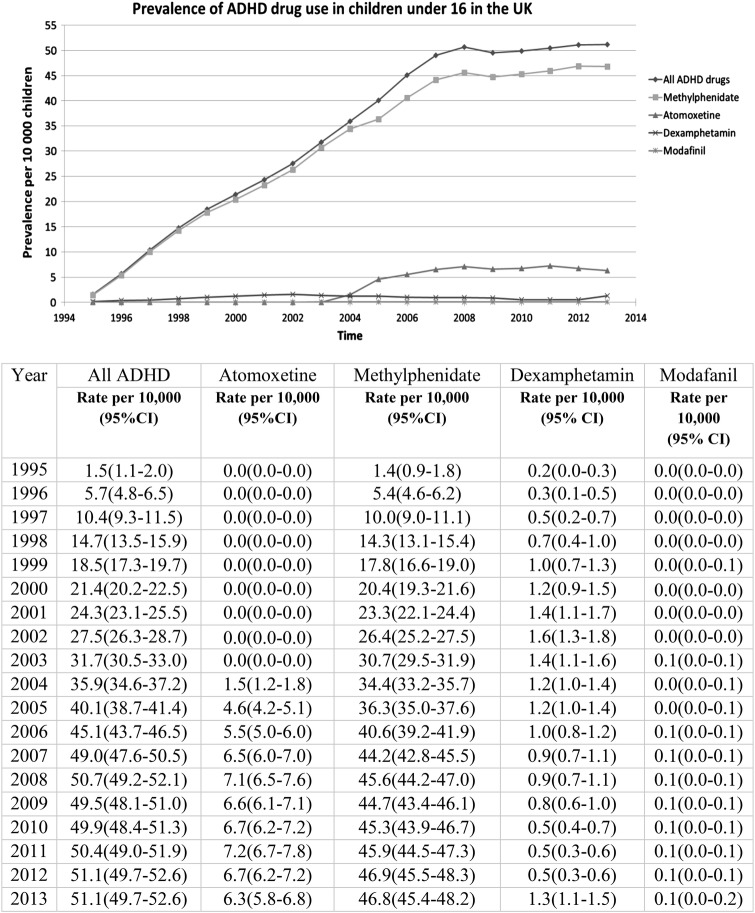
Annual prevalence rates of ADHD drugs per 10 000 children under sixteen between 1995 and 2013 in the CPRD database, N=14, 748. Denominators are mid-year counts of children under sixteen in the CPRD. ADHD, attention deficit and hyperactivity disorder; CPRD, Clinical Practice Research Datalink.

Since only 13 children in the database received ADHD drugs between 1992 and 1995, all further analyses begin in 1995. Between 1995 and 2013, overall prevalence of children receiving ADHD drugs increased 34-fold from 1.5/10 000 children to 50.7/10 000 children in 2008 and then stabilises to 51.1/10 000 children in 2013 (figure 1). Most of this increase is in methylphenidate although atomoxetine use increased nearly fivefold from 1.5 in 2004 to 7.1/10 000 children in 2008 and then decreased to 6.3/10 000 in 2013. Most of the increase in the prevalence of use of ADHD drugs was observed between 1995 and 2008 for methylphenidate and atomoxetine (test for trend in the methylphenidate rate after 2008 p=0.17; test for trend in the atomoxetine rate after 2008 p=0.09). Dexamfetamine and modafinil show relatively low use in children in the UK with respectively 1.3 and 0.1/10 000 children using these medications in 2013.

**Figure 2:**
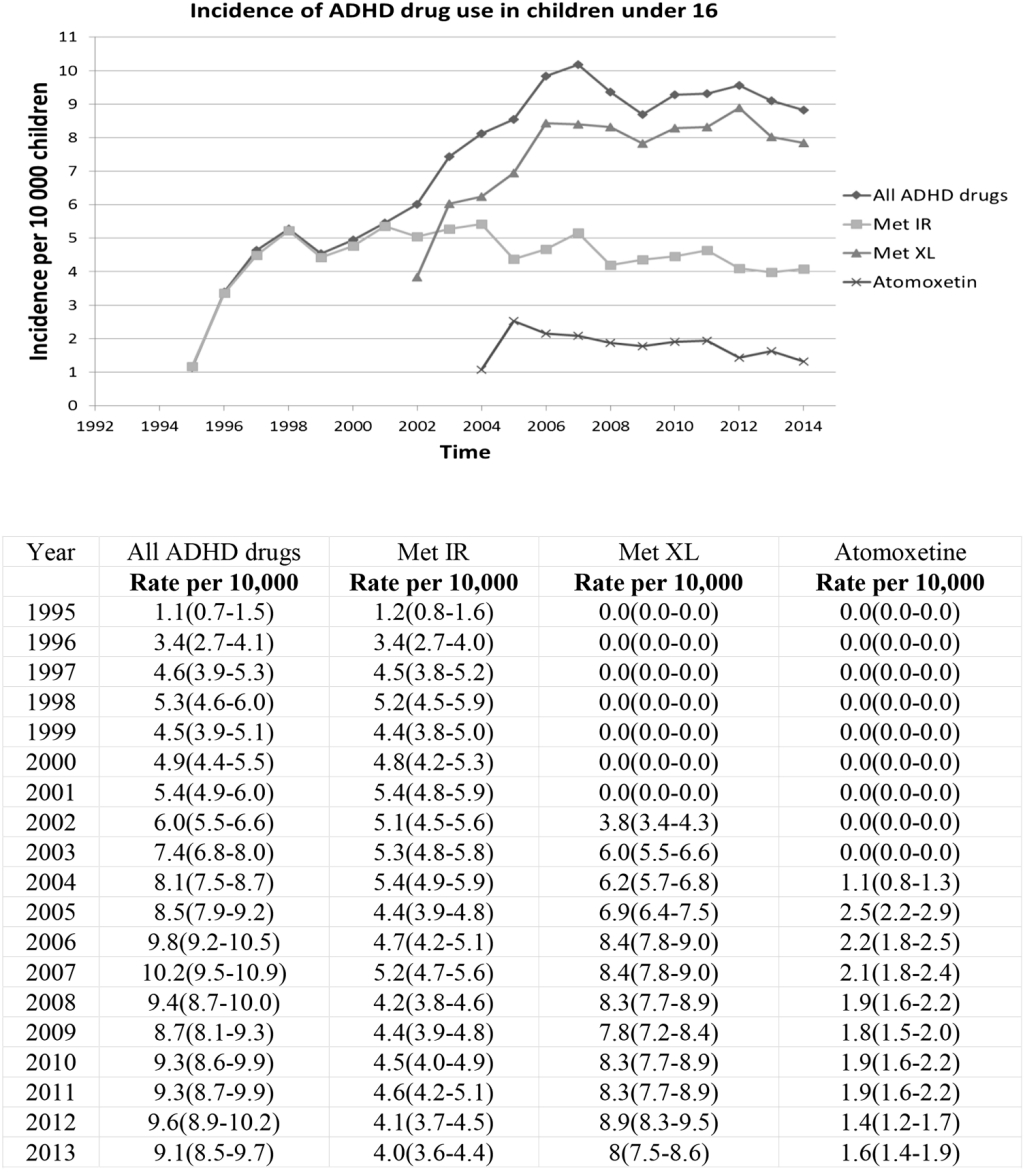
Incidence of ADHD drug initiation per 10 000 children under sixteen between 1995 and 2013 (N=10, 561) Met IR= immediate release; Met XL=extended release. ADHD, attention deficit and hyperactivity disorder.

We identified 10 566 children (84% boys) who had at least a year of registration in the CPRD before their first prescription of ADHD drug and a first prescription of ADHD drug between 1992 and 2013. Most children (58%) had their first prescription of ADHD medication between the age of 6 and 11 (primary school age), the majority (92%) receiving methylphenidate. The overall incidence of ADHD drug use increased ninefold between 1995 and 2013 from 1.1 to 9.1/10 000 children. However, incidence of new ADHD drugs use decreased slightly from 10.2 in 2007 to 9.1/10 000 in 2013. Again, this pattern is mainly driven by the use of methylphenidate. We can observe how methylphenidate ‘immediate release’ use is slowly substituted by ‘extended release’ methylphenidate after 2002, after its introduction in the UK (figure 2). Atomoxetine incidence more than doubled in the first 2 years of the marketing authorisation from 1.1/10 000 in 2004 to 2.5 in 2005 per 10 000 children in 2005 and then decreased to 1.6/10 000 children by 2013.

**Figure 3:**
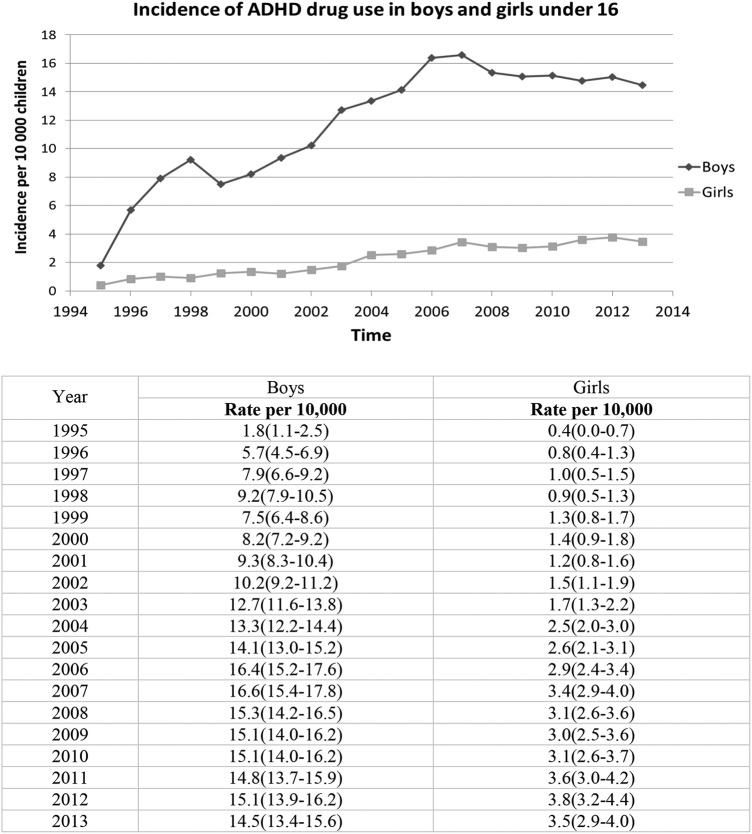
Gender specific incidence of methylphenidate treatment initiation in children under sixteen per 10, 000 children between 1995 and 2013, N=10, 561.

In boys, the incidence of methylphenidate use increased ninefold to reach 16.6/10 000 and decreased to 14.5 in 2013. In girls, the incidence increased ninefold to reach 3.4/10 000 in 2007 and then remained stable at 3.5/10 000 children until 2013 (figure 3). Although rates of use were much higher in boys, the proportionate increase was similar.

### Prescriptions in very young children/possible off-label use

Slightly more than 6% (659) of these children received 4123 prescriptions under the licensed age (6 years old in the UK) although 76% were issued between the ages of 5 and 6. Of the 659 children who had a recorded first prescription of an ADHD drug before age six, 245 (37%) had data missing on their month of birth. As a sensitivity analysis, we added 6 months to their age. We observed that 133 were still below six at the date of their first prescription of ADHD drug. Therefore, we can conclude that (659–245+133) 547 (4%) of children in our sample are likely to have been prescribed ADHD medication below age six. While dexamphetamine is licensed for children from 3 years only 52 children of 659 had a first prescription aged under 6, and only two under 3.

### Persistence of ADHD drug treatment in children under 16 in the UK

We identified 1115 (11%) children of 10 566 who had only one ADHD drug prescription and were registered in the database for more than 3 months after this prescription. These patients were not thought to be representative of the majority of children treated for ADHD and were therefore excluded from the retention analysis. This analysis was also split into three groups, under 6, 6–10 and 11–15 years.

**Figure 4:**
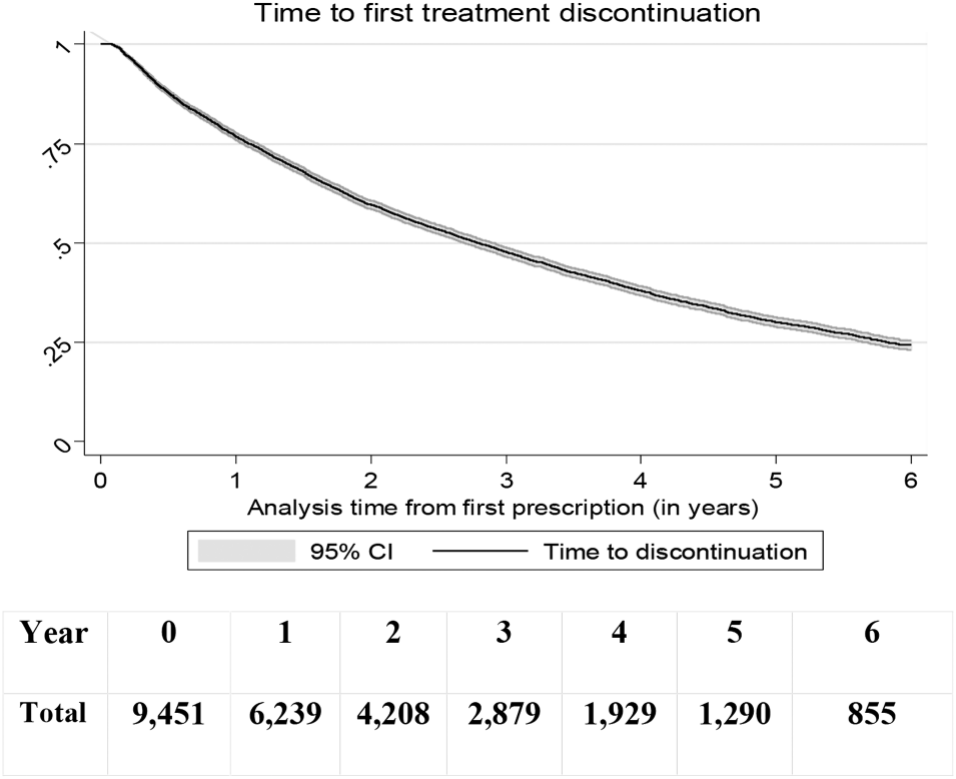
Time-to-discontinuation (90 days interruption) of all ADHD treatments in children, N=9,451. ADHD, attention deficit and hyperactivity disorder.

**Figure 5:**
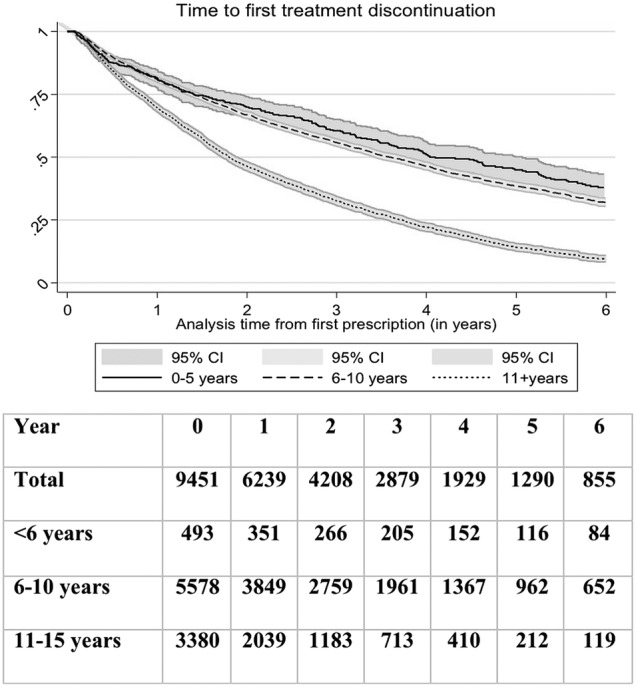
Time-to-discontinuation (90 days interruption) of all ADHD treatments in all children, according to the age of start of treatment, N=9,451. ADHD, attention deficit and hyperactivity disorder.

Our persistence analysis estimated time to the first discontinuation of the treatment (no ADHD drug at least 90 days after their prescription ended). At 1 year, 77% (95% CI 76% to 78%) of children were still taking ADHD drugs decreasing to 60% (59–61%) after 2 years. There was strong evidence that the probability of stopping ADHD treatments within 6 years was higher in older (11 to 15) compared to younger children (6–10), HR=1.87 (1.77 to 1.98) (see figures 4 and 5).

## Discussion

Overall prevalence of ADHD drug prescribing in children increased 34-fold and new prescribing of ADHD drugs increased eightfold between 1995 and 2013. However, the patterns of prevalent and incidence ADHD drug prescribing are changing.

Both prevalence and incidence rates started decreasing from 2007 to 2008 and remain stable up to 2013. Methylphenidate was by far the most used drug with 94% of children receiving it. Overall persistence on ADHD medication in children receiving >1 prescription was longer than in some countries. We found 77% (76–78%) of children were still under treatment after 1 year.

### UK rates among ADHD drug prescribing in Europe and the USA

First, it is important to note that UK rates of ADHD drug prescribing in children are ∼10 times lower than US rates (0.4% UK vs 4.4% in the USA in 2005),^16^ two to five times lower than Germany (0.49% in UK vs 2.21% in Germany in 2007 or 0.45% in UK vs0.9% in Germany in 2006)^17^ ^18^ and more than four times lower than in the Netherlands (4.5 vs 19.5/1000 in 2006).^6^ However, UK rates are twice as high as in France (4.0 UK in our study vs 1.8/1000 children in 2005).^19^

### Trends in ADHD drug prescribing in children, past and recent trends

Our analysis highlights two trends in UK ADHD drug prescribing in children. The first a strong increase after 1995, also observed in many other studies. Several studies reported a large increase between 1992 and 2008 in the prevalence of stimulant use in children under 18 in the UK.^20^ ^21^ In the USA, the prevalence of ADHD drug use increased from 2.8% to 4.4% between 2000 and 2005.^16^ France also reported rising rates of methylphenidate use between 2003 and 2005 with lower absolute prevalence than in the UK at 1.8/1000 children in 2005.^19^ In Germany, a 45% increase in prevalence of ADHD drugs in children under 18 years was observed between 2000 and 2007.^17^ Finally, a sevenfold increase of the use of stimulants was observed in the Netherlands between 1996 and 2006.^6^ Our study shows a clear break in the increasing trend of ADHD drug prescribing in children from 2007. Unfortunately, only few studies report rates of ADHD medication prescribing worldwide in children later than 2006. However, although an 1.84-fold increase in ADHD drug prescription was observed between 1995–1996 and 2003–2004 in the USA,^22^ the absolute rate of ADHD prescriptions changed little between 2002 and 2010 and the rate of methylphenidate use had no significant change between 2002 and 2010.^23^ In addition, a study investigating ADHD medication use in children in the US MEPS (medical expenditure's panel survey) shows a relatively stable rate with 3.43% of ADHD medication use in children in 2004 and 3.45% in 2008.^24^ In Germany, a recent study shows the rate of ADHD drug prescribing in children in DDD (defined daily doses) between 1990 and 2010 using large insurance company data. They showed a slight change in the curve from 2008 although accurate numbers are not reported.^25^ As expected, the prevalence and incidence of diagnosed ADHD in the UK seems to follow a similar pattern from 2007.^26^ Stable or decreasing trends in ADHD diagnosis were also reported in Denmark and Germany.^27^ ^28^

### Why such a sudden change in prescribing patterns?

Previously, the increase in use of ADHD medications was mainly explained by a better recognition of the issue of the disorders,^29^ a more positive image of its pharmacological treatments, longer treatments going on during adolescence and an expansion of use among girls.^30^ The change in the increasing trend of ADHD drug prescribing in the UK may be explained by the UK reaching a sufficient recognition of the ADHD condition and may mean that most children who need treatment are now reached. Such a decrease or a ‘plateau’ may also occur when a medication is under suspicion of severe adverse reaction as was observed for antidepressants after warnings on suicidal attempts in children was issued.^31^ However, the health warning on ADHD were issued only in Canada in 2006, and referred to Adderall a drug not marketed in the UK.^32^ In addition, risk warnings were not associated with a large decline in drug prescribing in the USA^24^ and further investigations on cardiac effects did not conclude there were major risks.^33^ ^34^ Such changes may also be due to the update of the National Institute for Health and Care Excellence (NICE) guidelines of 2008 stipulating that “Drug treatment is not indicated as the first-line treatment for all school-age children and young people with ADHD” only advocating the use of medication for children with recognised ADHD and severe impairments who had previously benefited from non-pharmacological therapeutic options.^35^ This recommendation is different from the recent clinical practice guidelines from the American Academy of Pediatrics from 2011 recommending that “school-aged children (6–11 years) should prescribe US Food and Drug administration approved medication for ADHD” possibly in addition to or instead of behavioural therapy, although favouring both interventions.^35^

### Persistence

Most studies in the literature define the persistence of ADHD drug as time from first prescription to the first discontinuation of 3–6 months. Winterstein *et al*^22^ reported that only 49.9% of children under 20 were still receiving ADHD therapy after 1 year and 17.2% were still taking ADHD drugs after 5 years in a sample of Medicaid registered children in the USA. In 2001, Schirm *et al* looked at duration of stimulant therapy in the Netherlands from first prescription to the first discontinuation of 180 days. Half of children had stopped their treatment after 20 months for those starting treatment between 1997 and 1999.^36^ In Germany, only 63.9% of boys were still taking ADHD drugs after 12 months (time to first discontinuation of 3 months). In our study, UK shows the highest estimates of persistence with 77% of children still taking ADHD drugs at 1 year, and even with those with a single prescription included it is still 66%. This could be due to the higher prevalence of use of ADHD medication in other countries, suggesting a possible different approach to medicating children in general. This may be because recommendations for ADHD management in the UK are more stringent. Our study shows substantially lower persistence of ADHD medication in the older age group. This observation is consistent with the findings that treatments may be prematurely discontinued in young adults despite the latest guidelines advising continuation of ADHD treatments as long as they are clinically effective.^37^

The CPRD has major strengths in the study of the use of medication in children.^38^ It allows for a recent, large, representative longitudinal cohort of children observing current UK practice.

We also acknowledge some weaknesses. First, the CPRD only registers prescriptions issued by the general practitioners and not actual drug dispensation or consumption by the patient, nor does it register prescriptions issued in secondary care by child psychiatrists and paediatricians. However, our intention was to report trends in prescribing patterns, and additionally the extensive repeat prescribing we saw suggests consumption. The lack of accurate information on the exact age of patients for 38% of children with month of birth missing and exact dates for none may have introduced imprecision in the calculation of the number of children that received prescriptions of ADHD drugs under the licensed age (6 years old for atomoxetine and methylphenidate). However, our sensitivity analysis showed that this phenomenon was unlikely to explain the majority of off-label prescriptions. Overall, off-label prescribing of ADHD drug in children under 6 years old appeared to be relatively low.

Our analysis of treatment persistence was based on elapsed time between first prescription and a gap of more than 3 months of ADHD drug prescription. It is possible that children with apparent treatment breaks may have received prescriptions from hospital, specialists or other institutions or even had suspended treatment during holidays. In the UK, specialists initiate ADHD prescriptions and continued prescribing and monitoring is typically performed under shared-care arrangements by General Practitioners as recommended by NICE.^34^ As compliance was not assessed, it is also possible that children's prescriptions may have lasted longer. It is therefore possible that incidence and persistence were underestimated.

## Conclusions

Among other countries such as the USA, the Netherlands and Germany, the UK shows one of the lowest estimates in ADHD drug prescribing rates in children. Although the prevalence and incidence of ADHD drug use in children have substantially increased during the past two decades, it seems that it may have reached a plateau recently. Looking at overall persistence in prescribing of ADHD drugs, children in the UK show the highest rates of ADHD drug persistence compared to other European countries and the US. These findings may suggest that UK prescribing practice restricts the choice of children receiving ADHD drugs ensuring a better follow-up of these children.

Our study indicates a turning point in the patterns of ADHD drug prescribing in children in the UK.

## References

[1] Wold Health Organisation. International Statistical Classification of Diseases and Related Health Problems 10th Revision Secondary International Statistical Classification of Diseases and Related Health Problems 10th Revision 1997.

[2] Polanczyk G, Rohde LA Epidemiology of attention-deficit/hyperactivity disorder across the lifespan. Curr Opin Psychiatry 2007;20:386–92. 10.1097/YCO.0b013e3281568d7a 17551354

[3] Klassen AF, Miller A, Fine S Health-related quality of life in children and adolescents who have a diagnosis of attention-deficit/hyperactivity disorder. Pediatrics 2004;114:e541–7. 10.1542/peds.2004-0844 15520087

[4] Brown RT, Amler RW, Freeman WS Treatment of attention-deficit/hyperactivity disorder: overview of the evidence. Pediatrics 2005;115:e749–57. 10.1542/peds.2004-2560 15930203

[5] Zito JM, Safer DJ, DosReis S Psychotropic practice patterns for youth: a 10-year perspective. Arch Pediatr Adolesc Med 2003;157:17–25. 10.1001/archpedi.157.1.17 12517190

[6] Trip AM, Visser ST, Kalverdijk LJ Large increase of the use of psycho-stimulants among youth in the Netherlands between 1996 and 2006. Br J Clin Pharmacol 2009;67:466–8. 10.1111/j.1365-2125.2009.03373.x 19371321 PMC2679111

[7] May DE, Kratochvil CJ Attention-deficit hyperactivity disorder: recent advances in paediatric pharmacotherapy. Drugs 2010;70:15–40. 10.2165/11530540-000000000-00000 20030423

[8] CPRD T. The CPRD website. Secondary The CPRD website. http://www.cprd.com/intro.asp

[9] Wood L, Martinez C The General Practice Research Database: role in pharmacovigilance. Drug Saf 2004;27:871–81. 10.2165/00002018-200427120-00004 15366975

[10] García Rodríguez LA, Pérez Gutthann S Use of the UK General Practice Research Database for pharmacoepidemiology. Br J Clin Pharmacol 1998;45:419–25. 10.1046/j.1365-2125.1998.00701.x 9643612 PMC1873548

[11] Jick H, Jick SS, Derby LE Validation of information recorded on general practitioner based computerised data resource in the United Kingdom. BMJ 1991;302:766–8. 10.1136/bmj.302.6779.766 2021768 PMC1669537

[12] Hollowell J The General Practice Research Database: quality of morbidity data. Popul Trends 1997;36–40. 9134574

[13] Herrett E, Thomas SL, Schoonen WM Validation and validity of diagnoses in the General Practice Research Database: a systematic review. Br J Clin Pharmacol 2010;69:4–14. 10.1111/j.1365-2125.2009.03537.x 20078607 PMC2805870

[14] Cuzick J A Wilcoxon-type test for trend. Stat Med 1985;4:87–90. 10.1002/sim.4780040112 3992076

[15] Cramer JA, Roy A, Burrell A Medication compliance and persistence: terminology and definitions. Value Health 2008;11:44–7. 10.1111/j.1524-4733.2007.00213.x 18237359

[16] Castle L, Aubert RE, Verbrugge RR Trends in medication treatment for ADHD. J Atten Disord 2007;10:335–42. 10.1177/1087054707299597 17449832

[17] Schubert I, Koster I, Lehmkuhl G The changing prevalence of attention-deficit/hyperactivity disorder and methylphenidate prescriptions: a study of data from a random sample of insurees of the AOK Health Insurance Company in the German State of Hesse, 2000–2007. Dtsch Arztebl Int 2010;107:615–21. 10.3238/arztebl.2010.0615 20948775 PMC2947846

[18] Knopf H, Holling H, Huss M Prevalence, determinants and spectrum of attention-deficit hyperactivity disorder (ADHD) medication of children and adolescents in Germany: results of the German Health Interview and Examination Survey (KiGGS). BMJ Open 2012;2:pii: e000477 10.1136/bmjopen-2011-000477 PMC3533105 23180453

[19] Knellwolf AL, Deligne J, Chiarotti F Prevalence and patterns of methylphenidate use in French children and adolescents. Eur J Clin Pharmacol 2008;64:311–17. 10.1007/s00228-007-0401-6 18026941

[20] Hsia Y, Maclennan K Rise in psychotropic drug prescribing in children and adolescents during 1992–2001: a population-based study in the UK. Eur J Epidemiol 2009;24:211–16. 10.1007/s10654-009-9321-3 19266290

[21] McCarthy S, Wilton L, Murray ML The epidemiology of pharmacologically treated attention deficit hyperactivity disorder (ADHD) in children, adolescents and adults in UK primary care. BMC Pediatr 2012;12:78 10.1186/1471-2431-12-78 22712630 PMC3472167

[22] Winterstein AG, Gerhard T, Shuster J Utilization of pharmacologic treatment in youths with attention deficit/hyperactivity disorder in Medicaid database. Ann Pharmacother 2008;42:24–31. 10.1345/aph.1K143 18042808

[23] Chai G, Governale L, McMahon AW Trends of outpatient prescription drug utilization in US children, 2002–2010. Pediatrics 2012;130:23–31. 10.1542/peds.2011-2879 22711728

[24] Barry CL, Martin A, Busch SH ADHD medication use following FDA risk warnings. J Ment Health Policy and Econ 2012;15:119–25. 23001280 PMC3896970

[25] Garbe E, Mikolajczyk RT, Banaschewski T Drug treatment patterns of attention-deficit/hyperactivity disorder in children and adolescents in Germany: results from a large population-based cohort study. J Child Adolesc Psychopharmacol 2012;22:452–8. 10.1089/cap.2012.0022 23234588 PMC3523251

[26] Holden SE, Jenkins-Jones S, Poole CD The prevalence and incidence, resource use and financial costs of treating people with attention deficit/hyperactivity disorder (ADHD) in the United Kingdom (1998 to 2010). Child Adolesc Psychiatry Ment Health 2013;7:34 10.1186/1753-2000-7-34 24119376 PMC3856565

[27] Schlack R, Mauz E, Hebebrand J [Has the prevalence of parent-reported diagnosis of attention deficit hyperactivity disorder (ADHD) in Germany increased between 2003–2006 and 2009–2012? Results of the KiGGS-study: first follow-up (KiGGS Wave 1)]. Bundesgesundheitsblatt Gesundheitsforschung Gesundheitsschutz 2014;57:820–9. 10.1007/s00103-014-1983-7 24950831

[28] Mohr Jensen C, Steinhausen HC Time trends in incidence rates of diagnosed attention-deficit/hyperactivity disorder across 16 years in a nationwide Danish registry study. J Clin Psychiatry 2015;76:e334–41. 10.4088/JCP.14m09094 25830455

[29] Sayal K, Ford T, Goodman R Trends in recognition of and service use for attention-deficit hyperactivity disorder in Britain, 1999–2004. Psychiatr Serv 2010;61:803–10. 10.1176/appi.ps.61.8.803 20675839

[30] Safer DJ, Zito JM, Fine EM Increased methylphenidate usage for attention deficit disorder in the 1990s. Pediatrics 1996;98 Pt 1):1084–8. 8951257

[31] Gibbons RD, Brown CH, Hur K Early evidence on the effects of regulators’ suicidality warnings on SSRI prescriptions and suicide in children and adolescents. Am J Psychiatry 2007;164:1356–63. 10.1176/appi.ajp.2007.07030454 17728420

[32] Food and Drug Administration F. Public Health Advisory for Adderall and Adderall XR. Secondary Public Health Advisory for Adderall and Adderall XR 2005 http://www.fda.gov/Drugs/DrugSafety/PostmarketDrugSafetyInformationforPatientsandProviders/ucm111441.htm

[33] Schelleman H, Bilker WB, Strom BL Cardiovascular events and death in children exposed and unexposed to ADHD agents. Pediatrics 2011;127:1102–10. 10.1542/peds.2010-3371 21576311 PMC3387871

[34] Cooper WO, Habel LA, Sox CM ADHD drugs and serious cardiovascular events in children and young adults. N Engl J Med 2011;65:1896–904. 10.1056/NEJMoa1110212 PMC4943074 22043968

[35] CG72 Attention deficit hyperactivity disorder (ADHD). NICE guideline National institute for health and clinical excellence, 2008.

[36] Schirm E, Tobi H, Zito JM Psychotropic medication in children: a study from the Netherlands. Pediatrics 2001;108:E25 10.1542/peds.108.2.e25 11483835

[37] Wong IC, Asherson P, Bilbow A Cessation of attention deficit hyperactivity disorder drugs in the young (CADDY)--a pharmacoepidemiological and qualitative study. Health Technol Assess 2009;13:iii–iv, ix-xi, 1–120. 10.3310/hta13490 19883527

[38] Helms PJ, Ekins Daukes S, Taylor MW Utility of routinely acquired primary care data for paediatric disease epidemiology and pharmacoepidemiology. Br J Clin Pharmacol 2005;59:684–90. 10.1111/j.1365-2125.2005.02404.x 15948933 PMC1884863

